# Supplementary material for: Exploring Metabolic Pathways of Anamorelin, a Selective Agonist of the Growth Hormone Secretagogue Receptor, via Molecular Networking
Source: Pharmaceutics. 2023 Nov 29;15(12):2700. doi: 10.3390/pharmaceutics15122700 (PMC10747546; doi:10.3390/pharmaceutics15122700)
Supplement: Supplementary file 1 [file pharmaceutics-15-02700-s001.zip › pharmaceutics-2682863-supplementary.pdf]

Supplementary data

Table S1. Accurate mass data of anamorelin and its metabolites in HLM. The table summarizes expected biotransformation based on retention time (RT) and characteristic fragment ions.

Compound	Type of biotransformation	Retention time (min)	Elemental comp. (exp.)	Theoretical mass (*m/z*)	Measured mass (*m/z*)	Error (ppm)
Anamorelin	Parent	17.7	$C_{31}H_{43}N_6O_3$	547.3391	547.3389	-0.3
			$C_{16}H_{26}N_3O$	276.207	276.2067	-1
			$C_{13}H_{16}NO$	202.1226	202.1224	-0.9
			$C_{12}H_{16}N$	174.1277	174.1275	-1.1
			$C_5H_9N_2O$	113.0709	113.0712	2.6
			$C_3H_{11}N_2$	75.0917	75.0924	9.3
			C_3H_8N	58.0658	58.0658	0
M1	Demethylation	16.9	$C_{30}H_{41}N_6O_3$	533.3235	533.3235	0
			$C_{15}H_{24}N_3O$	262.1914	262.191	-1.5
			$C_{15}H_{21}N_2O$	245.1648	245.1646	-0.8
			$C_{13}H_{16}NO$	202.1226	202.1222	-2
			$C_{12}H_{16}N$	174.1277	174.1276	-0.6
			$C_8H_{14}N_2O$	154.1101	154.1098	-1.9
			$C_5H_9N_2O$	113.0709	113.0712	2.7
			$C_3H_9N_2$	73.076	73.0768	10.9
			C_3H_8N	58.0658	58.066	3.4
M2	Demethylation + desaturation	17.6	$C_{30}H_{39}N_6O_3$	531.3078	531.3083	0.9
			$C_{15}H_{22}N_3O$	260.1757	260.1755	-0.8
			$C_{13}H_{16}NO$	202.1226	202.1223	-1.5
			$C_{12}H_{16}N$	174.1277	174.1277	0
			$C_8H_{13}N_2O$	153.1022	153.1022	0
			$C_5H_9N_2O$	113.0709	113.0712	2.7
			C_3H_8N	58.0658	58.066	3.4

Table S1. *Cont.*

Compound	Type of biotransformation	Retention time (min)	Elemental comp. (exp.)	Theoretical mass (*m/z*)	Measured mass (*m/z*)	Error (ppm)
M3a	Hydroxylation	15.8	$C_{31}H_{43}N_6O_4$	563.334	563.3372	5.7
			$C_{16}H_{26}N_3O$	276.207	276.2068	-0.7
			$C_{13}H_{16}NO$	202.1226	202.1224	-1
			$C_{12}H_{16}N$	174.1277	174.1276	-0.6
			$C_5H_9N_2O$	113.0709	113.0711	1.8
			$C_3H_{11}N_2$	75.0917	75.0924	9.3
			C_3H_8N	58.0658	58.066	3.4
			$C_9H_{10}ON$	148.0757	148.0756	-0.6
M3b	Hydroxylation	16.5	$C_{31}H_{43}N_6O_4$	563.334	563.3371	5.5
			$C_{16}H_{26}N_3O$	276.207	276.2071	0.3
			$C_{13}H_{16}NO$	202.1226	202.1225	-0.4
			$C_{12}H_{16}N$	174.1277	174.1277	0
			$C_5H_9N_2O$	113.0709	113.0711	1.7
			$C_3H_{11}N_2$	75.0917	75.0925	10.6
			C_3H_8N	58.0658	58.066	3.4
M4a	Dihydroxylation	15.2	$C_{31}H_{43}N_6O_5$	579.3289	579.3322	5.7
			$C_{16}H_{26}N_3O$	276.207	276.2071	0.4
			$C_{13}H_{16}NO$	202.1226	202.1226	0
			$C_{12}H_{16}N$	174.1277	174.1276	-0.6
			$C_3H_{11}N_2$	75.0917	75.0924	9.3
			C_3H_8N	58.0658	58.066	3.4
M4b	Dihydroxylation	15.4	$C_{31}H_{43}N_6O_5$	579.3289	579.3322	5.6
			$C_{16}H_{26}N_3O$	276.207	276.2068	-0.7
			$C_{13}H_{16}NO$	202.1226	202.1229	1.4
			$C_{12}H_{16}N$	174.1277	174.1277	0
			$C_3H_{11}N_2$	75.0917	75.0925	10.6
			C_3H_8N	58.0658	58.066	3.4

Table S1. *Cont.*

Compound	Type of biotransformation	Retention time (min)	Elemental comp. (exp.)	Theoretical mass (*m/z*)	Measured mass (*m/z*)	Error (ppm)
M4c	Dihydroxylation	15.8	$C_{31}H_{43}N_6O_5$	579.3289	579.3323	5.8
			$C_{16}H_{26}N_3O$	276.207	276.2076	2.1
			$C_{13}H_{16}NO$	202.1226	202.1226	0
			$C_{12}H_{16}N$	174.1277	174.1277	0
			$C_3H_{11}N_2$	75.0917	75.0925	10.6
			C_3H_8N	58.0658	58.0661	5.1
M5a	Hydroxylation + demethylation	14.9	$C_{30}H_{41}N_6O_4$	549.3183	549.319	1.3
			$C_{15}H_{24}N_3O$	262.1914	262.1908	-2.3
			$C_{15}H_{21}N_2O$	245.1648	245.1646	-0.8
			$C_{13}H_{16}NO$	202.1226	202.1223	-1.5
			$C_{12}H_{16}N$	174.1277	174.1276	-0.6
			$C_8H_{14}N_2O$	154.1101	154.1101	0
			$C_3H_9N_2$	73.076	73.0768	10.9
			C_3H_8N	58.0658	58.0661	5.2
			$C_9H_{10}ON$	148.0757	148.0751	-4.5
M5b	Hydroxylation + demethylation	15.6	$C_{30}H_{41}N_6O_4$	549.3183	549.3189	1
			$C_{15}H_{24}N_3O$	262.1914	262.1916	0.7
			$C_{15}H_{21}N_2O$	245.1648	245.1645	-1.2
			$C_{13}H_{16}NO$	202.1226	202.1223	-1.4
			$C_{12}H_{16}N$	174.1277	174.1278	0.5
			$C_3H_9N_2$	73.076	73.0768	10.9
			C_3H_8N	58.0658	58.0661	5.1

Table S1. *Cont.*

Compound	Type of biotransformation	Retention time (min)	Elemental comp. (exp.)	Theoretical mass (*m/z*)	Measured mass (*m/z*)	Error (ppm)
M5c	Hydroxylation + demethylation	15.9	$C_{30}H_{41}N_6O_4$	549.3183	549.319	1.2
			$C_{15}H_{24}N_3O$	262.1914	262.1911	-1.1
			$C_{15}H_{21}N_2O$	245.1648	245.165	0.8
			$C_{12}H_{16}N$	174.1277	174.1278	0.5
			$C_3H_9N_2$	73.076	73.0768	10.9
			C_3H_8N	58.0658	58.066	3.4
M6	Hydroxylation + desaturation	15.7	$C_{31}H_{41}N_6O_4$	561.3184	561.3214	5.3
			$C_{13}H_{16}NO$	202.1226	202.1225	-0.5
			$C_{12}H_{16}N$	174.1277	174.1277	0
			$C_5H_9N_2O$	113.0709	113.0712	2.7
			C_3H_8N	58.0658	58.066	3.4
M7	*N*-dealkylation	14.2	$C_{16}H_{26}N_3O$	276.207	276.2072	0.7
			$C_{13}H_{16}NO$	202.1226	202.1225	-0.5
			$C_{12}H_{16}N$	174.1277	174.1275	-1.1
			$C_3H_{11}N_2$	75.0917	75.0924	9.3
M8	*N*-dealkylation + demethylation	12.5	$C_{15}H_{24}N_3O$	262.1914	262.1916	0.8
			$C_{15}H_{21}N_2O$	245.1648	245.1647	-0.4
			$C_{13}H_{16}NO$	202.1226	202.1225	-0.5
			$C_{12}H_{16}N$	174.1277	174.1277	0
			$C_8H_{13}N_2O$	153.1022	153.1022	0
			$C_3H_9N_2$	73.076	73.0768	10.9

Table S1. *Cont.*

Compound	Type of biotransformation	Retention time (min)	Elemental comp. (exp.)	Theoretical mass (*m/z*)	Measured mass (*m/z*)	Error (ppm)
M9	*N*-dealkylation + demethylation + desaturation	13.6	$C_{15}H_{22}N_3O$	260.1757	260.1761	1.5
			$C_{12}H_{16}N$	174.1277	174.1276	-0.6
			$C_8H_{13}N_2O$	153.1022	153.1022	0
M10	*N*-oxidation	19.5	$C_{31}H_{43}N_6O_4$	563.334	563.3372	5.7
			$C_{16}H_{26}N_3O$	276.207	276.2068	-0.7
			$C_{13}H_{16}NO$	202.1226	202.1226	0
			$C_{12}H_{16}N$	174.1277	174.1276	-0.6
			$C_3H_{11}N_2$	75.0917	75.0924	9.3
M11a	*N*-oxidation + hydroxylation	16.6	$C_{31}H_{43}N_6O_5$	579.3289	579.3321	5.5
			$C_{16}H_{26}N_3O$	276.207	276.2071	0.3
			$C_{13}H_{16}NO$	202.1226	202.1226	0
			$C_{12}H_{16}N$	174.1277	174.1276	-0.5
			$C_3H_{11}N_2$	75.0917	75.0924	9.3
			C_3H_8N	58.0658	58.0661	5.1
M11b	*N*-oxidation + hydroxylation	16.9	$C_{31}H_{43}N_6O_5$	579.3289	579.3322	5.7
			$C_{16}H_{26}N_3O$	276.207	276.2069	-0.4
			$C_{15}H_{21}N_2O$	245.1648	245.1643	-2
			$C_{13}H_{16}NO$	202.1226	202.1226	0
			$C_{12}H_{16}N$	174.1277	174.1278	0.6
			$C_3H_{11}N_2$	75.0917	75.0924	9.3
			$C_3H_9N_2$	73.076	73.0768	10.9
M12	*N*-oxidation + demethylation	18.9	$C_{30}H_{41}N_6O_4$	549.3183	549.319	1.3
			$C_{15}H_{24}N_3O$	262.1914	262.1904	-3.8
			$C_{15}H_{21}N_2O$	245.1648	245.1646	-0.8
			$C_{13}H_{16}NO$	202.1226	202.1221	-2.5
			$C_{12}H_{16}N$	174.1277	174.1277	0

Table S1. *Cont.*

Compound	Type of biotransformation	Retention time (min)	Elemental comp. (exp.)	Theoretical mass (*m/z*)	Measured mass (*m/z*)	Error (ppm)
M13a*	+CO	16.7	$C_{32}H_{43}N_6O_4$	575.3345	575.3367	3.8
			$C_{16}H_{26}N_3O$	276.207	276.2068	-0.7
			$C_{13}H_{16}NO$	202.1226	202.1221	-2.5
			$C_{11}H_{11}N_2O$	187.0865	187.0865	0
			$C_{12}H_{16}N$	174.1277	174.1276	-0.6
			$C_3H_{11}N_2$	75.0917	75.0925	10.7
M13b*	+CO	18.0	$C_{32}H_{43}N_6O_4$	575.3345	575.3367	3.8
			$C_{16}H_{26}N_3O$	276.207	276.2074	1.4
			$C_{13}H_{16}NO$	202.1226	202.123	1.9
			$C_{12}H_{16}N$	174.1277	174.1276	-0.5
			$C_3H_{11}N_2$	75.0917	75.0925	10.6
M14a*	CO + oxidation	17.8	$C_{32}H_{43}N_6O_5$	591.3274	591.3317	7.2
			$C_{16}H_{26}N_3O$	276.207	276.2076	2.1
			$C_{13}H_{16}NO$	202.1226	202.1228	0.9
			$C_{12}H_{16}N$	174.1277	174.1277	0
			$C_3H_{11}N_2$	75.0917	75.0924	9.3
M14b*	CO + oxidation	19.1	$C_{32}H_{43}N_6O_5$	591.3274	591.3317	7.2
			$C_{16}H_{26}N_3O$	276.207	276.2069	-0.3
			$C_{13}H_{16}NO$	202.1226	202.1224	-0.9
			$C_{11}H_{11}N_2O$	187.0865	187.0864	-0.5
			$C_{12}H_{16}N$	174.1277	174.1275	1.1
M15a*	CO + demethylation	15.6	$C_{31}H_{41}N_6O_4$	561.3184	561.3218	6.0
			$C_{11}H_{11}N_2O$	187.0865	187.0863	-1.0
			$C_{15}H_{21}N_2O$	245.1648	245.1646	-0.8
			$C_3H_9N_2$	73.076	73.0769	12.3
M15b*	CO + demethylation	16.9	$C_{31}H_{41}N_6O_4$	561.3184	561.3213	5.1
			$C_{12}H_{16}N$	174.1277	174.1278	0.5
			$C_{15}H_{21}N_2O$	245.1648	245.1644	-1.6
			C_3H_8N	58.0658	58.0662	6.8
